# Risk factors for avascular necrosis of the femoral head after surgical treatment of developmental dysplasia of the hip in children: a systematic review and meta-analysis

**DOI:** 10.3389/fped.2026.1811138

**Published:** 2026-04-13

**Authors:** Zhuotao Guo, Xingguang Chen, Guoyu Dai, Mingfeng Xue

**Affiliations:** Department of Pediatric Orthopedic, The Second Affiliated Hospital of Jiaxing University, Jiaxing, China

**Keywords:** avascular necrosis, developmental dysplasia of the hip, meta-analysis, risk factors, systematic review

## Abstract

**Objective:**

This study aims to explore the risk factors for avascular necrosis (AVN) in children with developmental dysplasia of the hip (DDH) after surgical reduction through systematic review and meta-analysis.

**Methods:**

We searched PubMed, Embase, Cochrane Library, and Web of Science for studies investigating risk factors for postoperative AVN in children with DDH. The search was conducted up to May 1, 2025. Data were analyzed using Stata version 15.1.

**Results:**

A total of 16 studies involving 1,631 pediatric patients (1,941 hips) with DDH following surgery were included. Among them, AVN occurred in 468 hips. Multivariate meta-analysis showed absence of ossific nucleus [OR = 2.60, 95% CI (1.73, 3.91), *P* = 0.001], IHDI classification grade III/IV [OR = 2.43, 95% CI (1.46,4.03), *P* = 0.001] and secondary procedures [OR = 2.56, 95% CI (1.02, 6.46), *P* = 0.046] as independent risk factors for AVN after surgical treatment of DDH in children.

**Conclusion:**

Based on current evidence, higher grade dislocations, absence of ossific nucleus and secondary procedures are independent risk factors for AVN following surgical treatment of DDH in children.

**Systematic Review Registration:**

https://www.crd.york.ac.uk/PROSPERO/recorddashboard, PROSPERO CRD420251135105.

## 1 Introduction

As a condition that disrupts normal hip joint maturation in children, DDH represents a frequent orthopedic deformity. Recently, some scholars have calculated that the incidence rate is 1–10 per 1,000 newborns (1, 2). Treatment for DDH aims to achieve and maintain a concentric reduction of the femoral head within the acetabulum. It has been reported that closed or open reduction in children with DDH achieves a success rate of up to 90% (3). However, postoperative AVN is a common and serious complication that significantly impairs hip joint function and long-term outcomes in affected children (4, 5).

Given the often unacceptable consequences of AVN following DDH surgery in children, identifying its associated risk factors is crucial. Although numerous studies have investigated these risk factors, a clear consensus remains elusive. Earlier studies have suggested potential risk factors for AVN after DDH reduction, such as excessive hip abduction, open reduction, and absence of an ossific nucleus (6), yet recent research has yielded conflicting findings. While some studies suggested that ossific nucleus may lower AVN risk (7, 8), a systematic review by Sankar et al. found no such association (9). Regarding surgical approach, some investigators report a lower incidence of AVN with successful closed reduction compared to open reduction (10, 11), whereas others have observed a significantly higher AVN rate following closed reduction (5).

The exact etiology of AVN remains elusive, posing a significant challenge for both clinicians and affected children. Therefore, identifying the associated risk factors for AVN following surgery in children with DDH is of great importance. However, the risk factors for postoperative femoral head necrosis remain controversial across many studies. To consolidate current evidence for clinical application, this meta-analysis was conducted to synthesize available data on these risk factors.

## 2 Materials and methods

This systematic review adhered to the Preferred Reporting Items for Systematic Reviews and Meta-Analyses guidelines and its protocol extension (12). The protocol was prospectively registered (CRD420251135105).

### 2.1 Literature search

We systematically searched PubMed, Embase, the Cochrane Library, and Web of Science up to May 1, 2025, with MeSH and free-text terms (“DDH”, “AVN”, “risk factors”). The detailed strategy is in Supplementary Table S1.

### 2.2 AVN classification

We took the occurrence of AVN after reduction surgery in children with DDH as the outcome. Currently, the recognized classifications for AVN in children with DDH include Kalamchi-MacEwen classification, Bucholz-Ogden classification, and Salter classification.

#### 2.2.1 Kalamchi and MacEwen

Grade I: The ossific nucleus is mottled or fragmented but remains largely spherical, with minimal height loss or coxa magna (13). Grade II: Lateral physeal damage is present, leading to the development of coxa valga. Grade III: Central physeal damage occurs, resulting in pronounced coxa brevis and coxa magna. Grade IV: There is total damage to both the femoral head and the physis.

#### 2.2.2 Bucholz and Ogden

Grade I: Irregular ossification or hypoplasia confined to the femoral head; metaphysis is normal (14). Grade II: Lateral metaphyseal injury with subsequent development of coxa valga.Grade III: Complete metaphyseal involvement, leading to a shortened femoral neck and trochanteric overgrowth.Grade IV: Medial metaphyseal defect resulting in varus deformity of the proximal femur.

#### 2.2.3 Salter

(1) Failure of appearance of the ossific nucleus within 1 year after reduction, (2) failure of growth in the ossific nucleus within 1 year after reduction, (3) proximal femoral metaphyseal widening, (4) epiphyseal fragmentation, and (5) residual deformity of the femoral head/neck (15).

### 2.3 Inclusion and exclusion criteria

#### 2.3.1 Inclusion criteria

(1) The average age of all included children was less than 3 years old; (2) All children were diagnosed with DDH and had undergone closed reduction or open reduction; (3) All study outcomes used the AVN classification (Kalamchi and MacEwen、Bucholz and Ogden、Salter); (4) The shortest follow-up time in all studies was more than 1 year; (5) Study includes the results of the analysis of AVN risk factors.

#### 2.3.2 Exclusion criteria

(1) Reviews, letters, editorial comments, case reports, conference abstracts, adult studies, nonclinical studies, unpublished articles, and non-English articles; (2) Insufficient data or inability to fully extract outcome indicators were required for conducting the meta-analysis; (3) included patients with teratologic dislocations or neuromuscular disorders.

### 2.4 Data extraction

Two reviewers independently performed all screening and data extraction processes. Studies were initially screened by title and abstract, followed by full-text assessment of eligible articles. Any discrepancies were resolved by consensus with a senior third reviewer. Following the predefined criteria, we screened studies, extracted, and cross-checked relevant data (including first author, year, country, design, sample size, gender, mean age, AVN classification and average follow-up time). Considering the differences in DDH classification among each study, we classified IHDI and Tönnis classification into the high-dislocation group (III/IV) and the low-dislocation group (I/II) for analysis.

### 2.5 Quality evaluation

The quality of included case-control studies was assessed using the Newcastle-Ottawa Scale (NOS) (16). This tool rates eight items across three domains: selection of study groups (4 points), comparability of groups (2 points), and ascertainment of exposure or outcome (3 points), yielding a maximum score of 9. Studies were classified as low (≤4 points), moderate (5–6 points), or high (≥7 points) quality based on their total NOS scores. We employed a dual independent review process with arbitration to resolve discrepancies.

### 2.6 Statistical analysis

We performed statistical analyses using Stata (version 15.1). For each included study, effect sizes were expressed as odds ratios (ORs) or standardized mean differences (SMDs), along with their corresponding 95% confidence intervals (CIs). Statistical heterogeneity was assessed using Cochran's *Q* test and the I² statistic. We applied a fixed-effects or random-effects model based on whether heterogeneity was low (I² ≤ 50%) or high (I² > 50%). The pooled OR was calculated based on the selected model. For analyses with I² > 50%, sensitivity was assessed using the leave-one-out method. Publication bias was evaluated using Egger's and Begg's tests, with statistical significance defined as *P* < 0.05.

## 3 Results

### 3.1 Results of the literature retrieval

**Figure 1:**
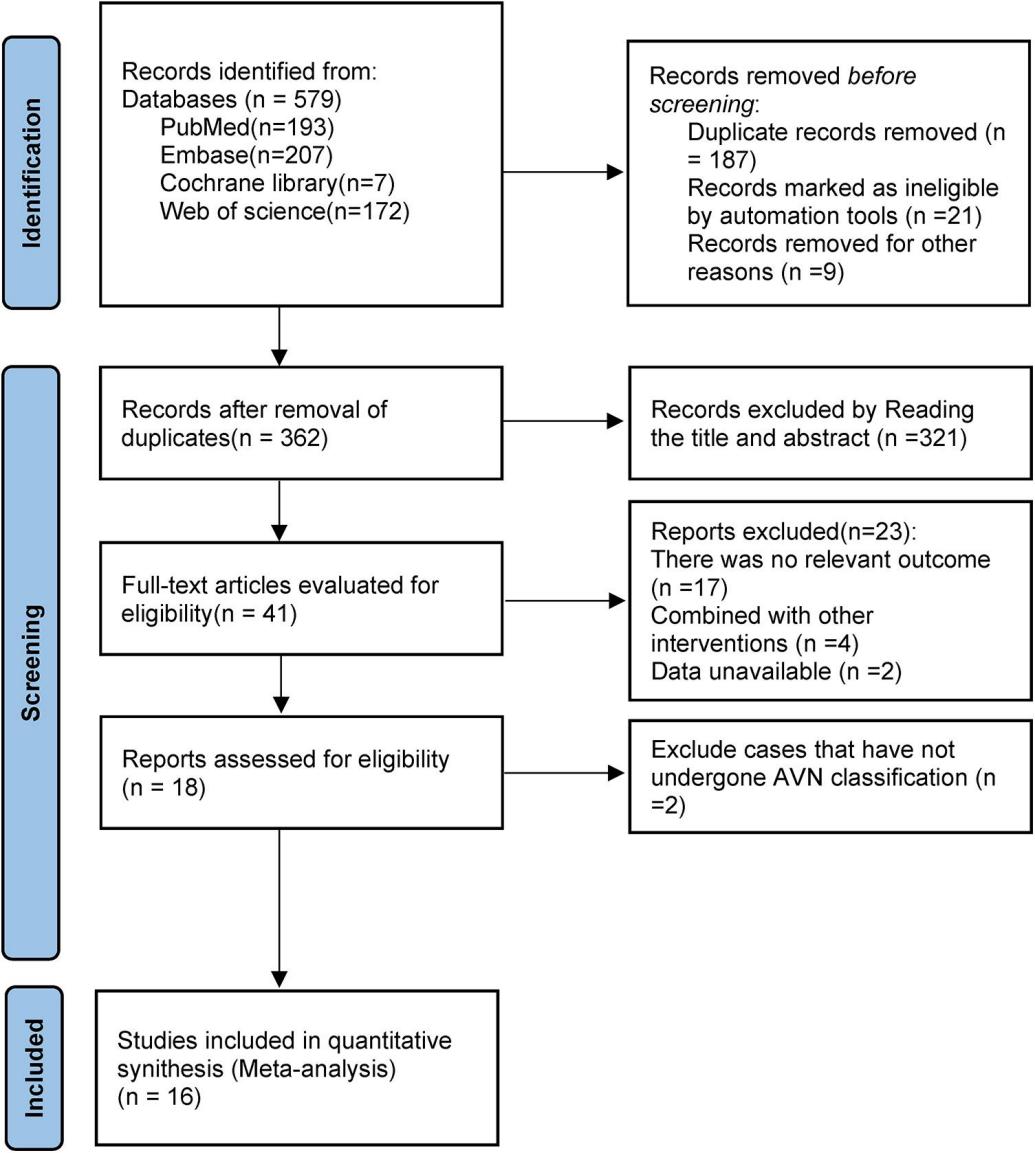
Flow chart of the literature search.

Searches across PubMed, Embase, the Cochrane Library, and Web of Science yielded 579 records. Following deduplication, 362 articles were screened by title/abstract, filtered to 41 for full-text assessment, and 16 studies were finally included. The selection flow is shown in Figure 1.

### 3.2 Basic features of the included literature

The 16 included studies (17–32) were all of the retrospective cohort study type. They enrolled 1,631 children (1,941 hips) following DDH surgery, with postoperative AVN occurring in 468 hips. The specific file characteristics can be found in Table 1. Sixteen articles were evaluated using the NOS criteria: one (24) scored 6 points, indicating that the research quality was moderate. The remaining articles scored 7–9 points, and the overall quality of those articles was high. See Table 2 for specific quality evaluations.

**Table 1:** Table of literature characteristics.

Study	Country	Study design	Sample size		Gender (M/F)	Avascular necrosis(hips)	Mean Age (months)	AVN classification	Average follow-up time(year)
			*N*	NH					
Ağuş (24)	Turkey	RCS	54	67	NA	20	11.9 (3–18)	Kalamchi-MacEwen	3.7 ± 1.2
Apostolides (28)	UK	RCS	76	79	8/68	16	13.8 (3–39)	Kalamchi-MacEwen	8.9 (0.5–16.5)
Bian (29)	China	RCS	108	140	8/100	44	16.6 ± 3.6	Salter/Kalamchi-MacEwen	10.1 (7–16)
Bozkurt (30)	Turkey	RCS	68	97	13/55	21	8 (4–12)	Bucholz Ogden	2.5 (2–4.5)
Cheon (19)	Korea	RCS	58	77	5/53	13	10.95 ± 4.7	Salter/Kalamchi-MacEwen	5.7 ± 2.3
JP Wu (20)	China	RCS	78	83	15/63	32	16.6 (4.3–33.5)	Kalamchi-MacEwen	1.8 (1–2.9)
Kheiri (21)	Iran	RCS	71	89	8/63	18	12.5 ± 3.9	Bucholz Ogden	≥3.0
Liu (27)	China	RCS	55	59	5/55	8	14.4 (6–28)	Kalamchi-MacEwen	2.2 (1–3.5)
Pang(OR) (31)	China	RCS	119	119	17/102	24	20.97 ± 6.03	Kalamchi-MacEwen	≥2.0
Pang(CR) (31)	China	RCS	92	92	8/84	15	10.97 ± 3.55	Kalamchi-MacEwen	≥2.0
Pospischill (25)	Austria	RCS	64	78	12/52	31	NA	Bucholz Ogden	6.8 (3.2–11.5)
QJ Wu (32)	China	RCS	254	278	NA	89	31.2	Kalamchi-MacEwen	3.8 ± 1.5
Roposch (17)	UK	RCS	89	105	15/74	37	8.96 (1.6–17.8)	Bucholz Ogden	9.1 (3–17.6)
Schur (26)	USA	RCS	70	82	8/62	29	10 (1–31)	Salter	5 (2–19)
Tang (22)	China	RCS	134	169	11/123	42	10.7 (4–22)	Kalamchi-MacEwen	3.19 ± 2.25
Zamzam (26)	SAU	RCS	131	189	16/115	23	18 (15–25)	Kalamchi-MacEwen	2.98
Zhang (18)	China	RCS	110	138	17/63	6	16.6 (6.4–33.2)	Bucholz Ogden	4.3(2–6.6)

N, Number of DDH; NH, The number of DDH hips; NA, Not applicable.

**Table 2:** NOS scores.

Study	Representativeness of the exposed group	Selection of non-exposed groups	Determination of exposure factors	Identification of outcome indicators not yet to be observed at study entry	Comparability of exposed and unexposed groups considered in design and statistical analysis	design and statistical analysis	Adequacy of the study's evaluation of the outcome	Adequacy of follow-up in exposed and unexposed groups	Total scores
Ağuş (24)	*	*	*	*	*	*	/	/	6/9
Apostolides (28)	*	*	*	*	*	*	*	*	8/9
Bian (29)	*	*	*	*	*	*	/	*	7/9
Bozkurt (30)	*	*	*	*	*	*	/	*	7/9
Cheon (19)	*	*	*	*	*	*	*	/	7/9
JP Wu (20)	*	*	*	*	*	*	*	/	7/9
Kheiri (21)	*	*	*	*	**	*	*	*	9/9
Liu (27)	*	*	*	*	*	*	*	/	7/9
Pang (31)	*	*	*	*	*	*	*	*	8/9
Pospischill (25)	*	*	*	*	*	*	*	*	8/9
QJ Wu (32)	*	*	*	*	*	*	*	*	8/9
Roposch (17)	*	*	*	*	*	*	/	*	7/9
Schur (26)	*	*	*	*	*	*	/	*	7/9
Tang (22)	*	*	*	*	*	*	*	/	7/9
Zamzam (26)	*	*	*	*	**	*	*	*	9/9
Zhang (18)	*	*	*	*	*	*	/	*	7/9

* or / indicates the evaluation of the indicators. * indicates compliance, / indicates non-compliance.

### 3.3 Single-factor meta-analysis

#### 3.3.1 Age

**Figure 2:**
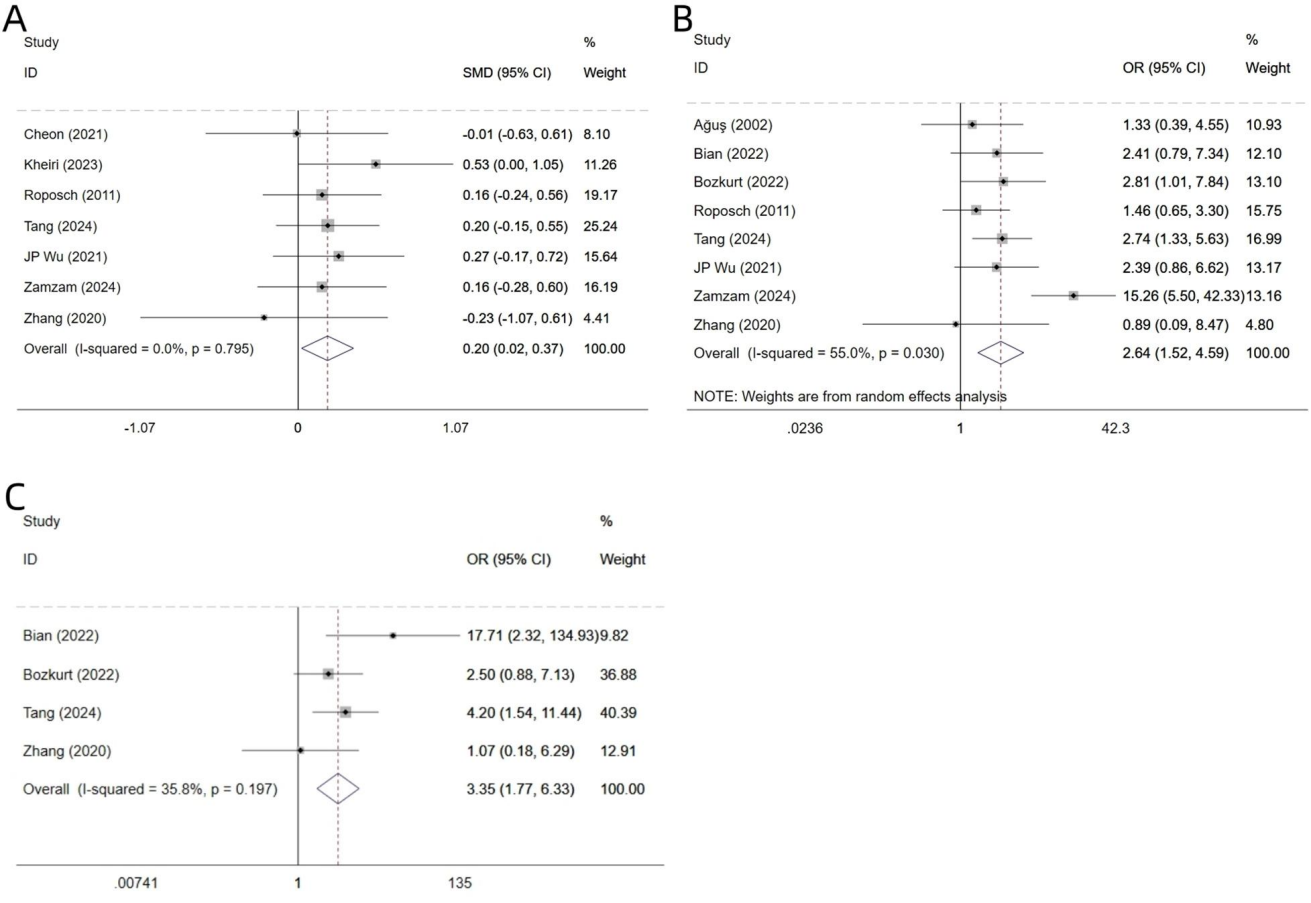
Forest plot of single-factor meta-analysis age **(A)**, ossific nucleus **(B)** and IHDI classification **(C)**.

Data on age were reported in seven studies (17–23). As heterogeneity was non-significant (I² = 0.0%, *P* = 0.798), a fixed-effects model was therefore employed for the meta-analysis. The pooled results revealed that older age was a statistically significant risk factor for AVN following surgical treatment of DDH in children [SMD = 0.20, 95% CI (0.02, 0.37), *P* = 0.026] (Figure 2A, Table 3).

**Table 3:** Single factor meta-analysis.

Risk factors	No of study	heterogeneity		SMD/OR (95%CI)	*P*	Egger	Begg
		I^2^(%)	*P*				
Age	7	0	0.798	0.20 (0.02, 0.37)	0.026	0.372	0.453
Male	9	0	0.726	1.05 (0.70, 1.58)	0.806	0.144	0.175
Bilateral	5	0	0.913	1.16 (0.74, 1.84)	0.517	0.142	0.232
Right	7	0	0.669	0.80 (0.59, 1.09)	0.153	0.176	0.779
IHDI grade	4	35.8	0.197	3.35 (1.77, 6.33)	0.001	1.000	0.806
Tönnis grade	4	0	0.426	1.82 (0.98, 3.39)	0.057	0.497	0.592
Ossific nucleus	8	55.0	0.030	2.64 (1.52, 4.59)	0.001	0.621	0.757
Adductor & iliopsoas tenotomy	6	0	0.438	1.49 (0.84, 2.64)	0.169	0.091	0.065
Abduction brace history	5	0	0.859	1.09 (0.69, 1.72)	0.708	0.425	0.624
Preop acetabular index	3	0	0.979	0.17(−0.03, 0.37)	0.087	0.602	0.737
Surgical approach	3	0	0.415	1.29 (0.90, 1.85)	0.072	0.523	0.497

#### 3.3.2 Ossific nucleus

Eight studies (17, 18, 20, 22–24, 29, 30) reported on ossific nucleus. Based on evidence of significant heterogeneity (I² = 55.0%, *P* = 0.030), we applied a random-effects model. The analysis demonstrated that absence of ossific nucleus was a risk factor for AVN following surgery for DDH in children, with a statistically significant difference [OR = 2.64, 95% CI (1.52, 4.59), *P* = 0.001] (Figure 2B, Table 3).

#### 3.3.3 IHDI classification

Four studies (18, 22, 29, 30) reported on the IHDI classification. Heterogeneity testing showed I² = 35.8% and *P* = 0.197; thus, fixed-effects model was used. The analysis indicated that IHDI classification (type III/IV) was a risk factor for AVN after surgery for DDH in children, with a statistically significant difference [OR = 3.35, 95% CI (1.77, 6.33), *P* = 0.001] (Figure 2C, Table 3). In addition, the funnel plot showed no significant publication bias for the above three indicators (Figures 4A–C).

#### 3.3.4 Other results from univariate meta-analyses

The univariate analysis in this study indicated that sex, unilateral/bilateral involvement, affected side, Tönnis classification, adductor tenotomy, history of preoperative abduction bracing, preoperative acetabular index (AI) angle, and surgical approach showed no statistically significant association with the development of AVN after surgery for DDH in children (Table 3).

### 3.4 Multi-factor meta-analysis results

#### 3.4.1 Ossific nucleus

**Figure 3:**
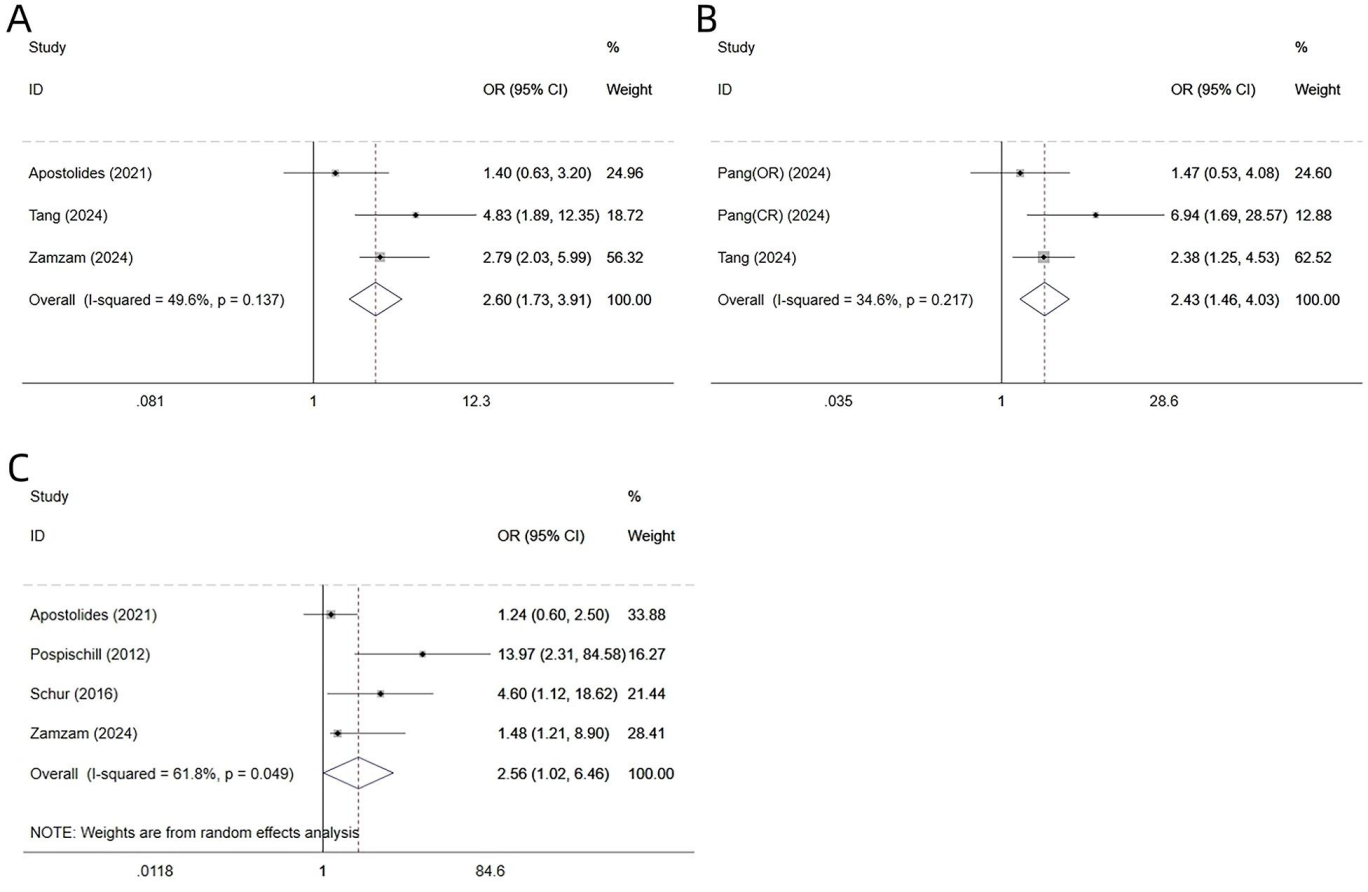
Forest plot of multivariate-factor meta-analysis ossific nucleus **(A)**, IHDI classification **(B)** and secondary procedures **(C)**.

Three studies (22, 23, 28) reported on the ossific nucleus. Heterogeneity testing revealed I² = 49.6% and *P* = 0.137; thus, a fixed-effects model was used. We found that the absence of an ossific nucleus was an independent risk factor for AVN after surgical treatment of DDH in children [ES = 2.60, 95% CI 1.73–3.91, *P* = 0.001] (Figure 3A, Table 4).

**Table 4:** Multi-factor meta-analysis.

Risk factors	No of study	heterogeneity		ES (95%CI)	*P*	Egger	Begg
		I^2^ (%)	*P*				
Age	7	36.7	0.148	−0.00 (−0.07, 0.06)	0.921	0.099	0.085
Male	6	14.8	0.319	0.42 (−0.21, 1.06)	0.189	0.188	0.374
IHDI grade	3	34.6	0.217	2.43 (1.46, 4.03)	0.001	0.602	0.694
Ossific nucleus	3	49.6	0.137	2.60 (1.73, 3.91)	0.001	0.963	0.602
Abduction angle	3	88.0	0.001	1.20 (−0.26, 2.66)	0.109	0.089	0.117
Secondary procedures	4	61.8	0.049	2.56 (1.02, 6.46)	0.046	0.077	0.089

#### 3.4.2 IHDI classification

Two studies (22, 31) reported on IHDI classification. Among these, Pang et al. (31) presented data separately for closed reduction and open reduction groups; we therefore analyzed these as two distinct subgroups. Heterogeneity testing showed I² = 34.6% and *P* = 0.217; accordingly, a fixed-effects model was applied. The analysis indicated that higher IHDI classification (type III/IV) was an independent risk factor for AVN after surgery for DDH in children [ES = 2.43, 95% CI (1.46, 4.03), *P* = 0.001] (Figure 3B, Table 4).

#### 3.4.3 Secondary procedures

**Figure 4:**
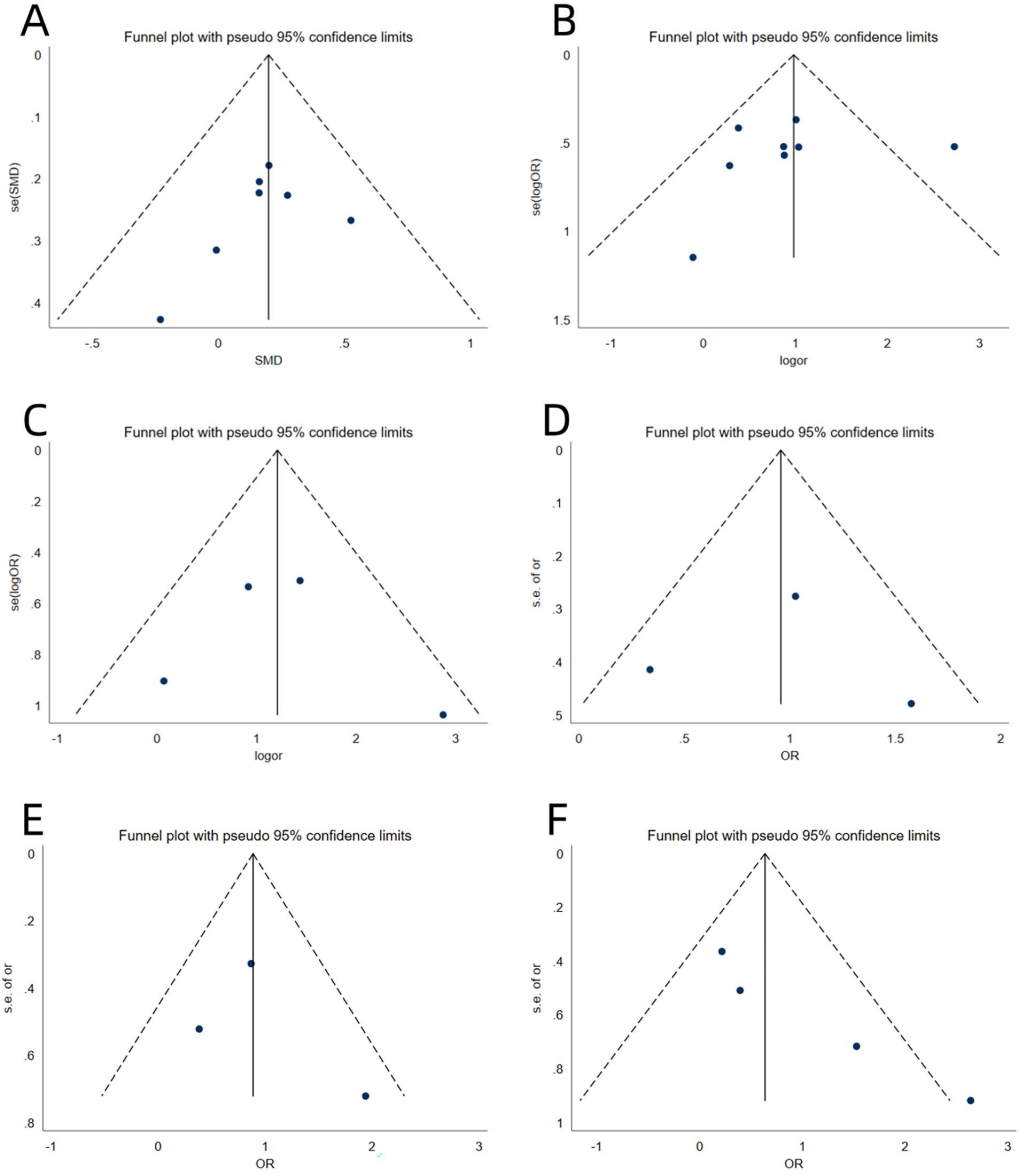
Funnel plot of meta analysis single-factor of age **(A)**, single-factor of ossific nucleus **(B)**, single-factor of IHDI classification **(C)**, multivariate-factor of ossific nucleus **(D)**, multivariate-factor of IHDI classification **(E)** and multivariate-factor of secondary procedures **(F)**.

Four studies (23, 25, 26, 28) reported on secondary procedures. Heterogeneity testing showed I² = 61.8% and *P* = 0.049; accordingly, a random-effects model was used. The analysis indicated that secondary procedures was an independent risk factor for AVN after surgery for DDH in children, with a statistically significant difference [ES = 2.56, 95% CI (1.02, 6.46), *P* = 0.046] (Figure 3D, Table 4). Funnel-plot analysis revealed no significant publication bias for these three variables (Figures 4D–F).

#### 3.4.4 Other multivariable meta-analysis results

Other multivariable results from this study indicate that age, sex, preoperative traction and the preoperative AI angle show no statistically significant association with postoperative AVN in children with DDH (Table 4).

## 4 Sensitive analysis

In the univariate analysis, the heterogeneity of the ossific nucleus indicator was found to be I² > 50%. A leave-one-out sensitivity analysis was performed. After excluding the ossific nucleus data from Zamzam et al., the statistical significance of the results changed [OR = 1.57, 95% CI (1.26, 1.97), *P* = 0.001], and heterogeneity was reduced (I² = 35.5%, *P* = 0.157). In the multivariable analysis, the heterogeneity of secondary procedures indicator was I² > 50%. A corresponding sensitivity analysis indicated that after excluding the study by Pospischill et al., heterogeneity diminished (I² = 25.4%, *P* = 0.262), and the previously significant effect disappeared [ES = 0.51, 95% CI (−0.14, 1.16), *P* = 0.124]. These results suggested that the above-mentioned studies were the primary sources of heterogeneity for these parameters.

## 5 Publication bias

Publication bias for each risk factor was evaluated with Egger's and Begg's tests. The results showed that in both univariate and multivariate analyses, all *P*-values exceeded 0.05, indicating no significant publication bias (Tables 3, 4).

## 6 Discussion

Although some previous studies (33, 34) had analyzed the risk factors for AVN or failure following reduction surgery in children with DDH, they were limited by their surgical approaches or their conclusions differed from those of earlier research. Incorporating recent advances in the field, we conducted a systematic review and meta-analysis of the risk factors for avascular necrosis of the femoral head after reduction surgery in children with developmental dysplasia of the hip. The study found that higher grade dislocations, absence of ossific nucleus and secondary procedures were independent risk factors for AVN in children with DDH.

The univariate analysis in this study revealed that older age was correlated with a higher risk of AVN. Previous studies have identified older age as a significant risk factor for AVN following both closed reduction (35, 36) and open reduction (32, 37). Older children tended to exhibit more severe pathological changes around hip joint, often necessitating more extensive and invasive surgical procedures to achieve anatomical reduction. Such interventions might directly compromise the fragile blood supply to the femoral head, which could partly explain why older age is considered a risk factor. However, several studies reported no association between age and AVN risk (8, 18, 19). Consistent with these findings, the multivariate logistic regression model in this study demonstrated that age was not an independent risk factor for AVN after controlling for other influencing variables. This discrepancy might be attributable to insufficient adjustment for confounding factors or the lack of detailed subgroup analyses in earlier studies.Age was found to be correlated with the presence of ossific nucleus, and some researchers have suggested that closed reduction was more appropriate after ossification of the femoral head has occurred (18, 38). Wirth et al. (39) proposed that early intervention in children under six months of age might increase the incidence of AVN, potentially acting as a confounding factor. Conversely, other investigators have proposed that early intervention in the presence of an ossific nucleus may serve as a protective factor (40).Additionally, some authors have noted that early or timely open reduction in older children with DDH may yield superior outcomes and reduce the risk of AVN compared with closed reduction (25). The findings of this study indicated that age does not constitute a significant independent risk factor for postoperative AVN. Therefore, regardless of age, early detection and timely intervention, along with the formulation of individualized treatment plans, are recommended for children with DDH.

In this study, the International Hip Dysplasia Institute (IHDI) and Tönnis classifications were used to differentiate the degree of dislocation in DDH. Patients were dichotomized into high- (Grade III/IV) and low-dislocation (Grade I/II) groups. The analysis showed that a higher degree of hip dislocation was linked to a higher risk of postoperative AVN—a result that remained consistent in the multivariate analysis. Pang et al. (31) indicated that the likelihood of developing AVN in IHDI grade III/IV is 6.94 times higher than in grade I/II. A high degree of dislocation reflects prolonged impairment of the femoral head blood supply, in which sustained traction on the lateral epiphyseal artery contributes to reduced perfusion (30, 41, 42). To reduce a high-dislocation femoral head, surgical intervention often requires extensive release of contracted soft tissues (such as the adductor muscles, iliopsoas muscle, and joint capsule). During this process, critical periarticular vessels are highly susceptible to traction or direct injury. Ramo et al. (43) suggested that higher Tönnis or IHDI classifications can predict an increased trend in AVN incidence. Similarly, Liu et al. (44) reached analogous conclusions and proposed that open reduction may reduce the risk of AVN in children with DDH who are 18 months or older and classified as IHDI grade III/IV. Our univariate analysis revealed no significant association between Tönnis classification and AVN. In contrast, IHDI classification demonstrated a more balanced distribution, with the majority of cases concentrated in Grades II/III (45). This more even distribution makes IHDI classification more discriminative in assessing the severity of dislocation, allowing it to better reflect the gradient of pathological changes and potentially offering a clinical advantage.

Both univariate and multivariate analyses identified absence of ossific nucleus as a risk factor for AVN, while its presence was considered protective (46). A related theory suggested that ossific nucleus may shield the femoral head from ischemic compression (47). The ossific nucleus is the first ossification center to appear within the femoral head, typically emerging 4–6 months after birth. Its presence marks the beginning of the transition from cartilage to hardened bone in the femoral head. As the ossific nucleus enlarges, it progressively forms the central structure supporting weight-bearing and growth of the femoral head (46, 48). Yilar et al. (40)observed that the presence of ossific nucleus provided a protective effect against AVN in children over 6 months of age, while no such additional protective effect was seen in children younger than 6 months. Some studies have found that children without ossific nucleus tend to develop more severe grades of AVN (7, 49). For DDH patients over 6 months of age without ossific nucleus, this indicated potential severe ischemia and an unfavorable biological environment in the femoral head, thereby increasing the risk of AVN. Some researchers (46, 50) advocated delaying closed reduction until the ossific nucleus is visible, considering its presence a protective factor against AVN. Additionally, the absence of an ossific nucleus poses challenges for reduction, as it complicates intraoperative fluoroscopic assessment of femoral head position and reduction quality, potentially increasing procedural difficulty and uncertainty. While some scholars have studied ossific nucleus volume using MRI, recent research (31) found that ossific nucleus size is not a significant risk factor for postoperative AVN in children with DDH, regardless of whether closed or open reduction is performed. Other recent studies (19) suggest that contrast-enhanced MRI of the femoral head after closed reduction can effectively predict the risk of subsequent AVN.

Multivariate analysis in this study revealed that secondary surgery increased the risk of AVN. The increased risk of AVN associated with secondary surgery may be attributed to factors such as deterioration of the hip joint environment, altered anatomical structures from the initial surgery, fragile blood supply to the femoral head, and more complex pathological changes. Some scholars indicated that patients who undergo secondary surgery have a 14-fold higher risk of developing AVN compared to those who do not (25). Secondary surgery remains a significant challenge for surgeons.

This study has several limitations. First, the number and diversity of included studies and outcome measures are limited, with most originating from China, potentially introducing selection bias. Second, a potential source of the observed heterogeneity is the variation in diagnostic criteria for DDH and AVN across studies. Third, some researchers currently consider Grade II and above as clinically significant AVN; however, some studies included in this analysis did not provide a detailed subclassification of AVN, which may introduce a certain degree of selection bias. Fourth, the analysis does not distinguish between ORs, risk ratios (RRs), and hazard ratios (HRs). Although the practical differences among these measures may be small, they assess disease risk in fundamentally different ways, which could introduce some bias into the results.

## 7 Conclusion

Based on the current evidence, higher grade dislocations, absence of ossific nucleus and secondary procedures are independent risk factors for AVN following surgery in children with DDH. Clinicians can use these indicators to facilitate early detection, diagnosis, and intervention for children with DDH, thereby reducing the risk of postoperative AVN.

## Data Availability

The original contributions presented in the study are included in the article/Supplementary Material, further inquiries can be directed to the corresponding author/s.

## References

[1] Kotlarsky P Haber R Bialik V Eidelman M. Developmental dysplasia of the hip: what has changed in the last 20 years? World J Orthop. (2015) 6(11):886–901. 10.5312/wjo.v6.i11.886 26716085 PMC4686436

[2] Murphy RF Kim YJ. Surgical management of pediatric developmental dysplasia of the hip. J Am Acad Orthop Sur. (2016) 24(9):615–24. 10.5435/JAAOS-D-15-00154 27509038

[3] Wang Y. Current concepts in developmental dysplasia of the hip and total hip arthroplasty. Arthroplasty. (2019) 1(1):2. 10.1186/s42836-019-0004-6 35240757 PMC8787940

[4] Domzalski M Synder M. Avascular necrosis after surgical treatment for developmental dysplasia of the hip. Int Orthop. (2004) 28(2):65–8. 10.1007/s00264-003-0522-1 15274235 PMC3474479

[5] Al Faleh AF Jawadi AH Al Sayegh S Al Rashedan BS Al Shehri M Al Shahrani A. Avascular necrosis of the femoral head: assessment following developmental dysplasia of the hip management. Int J Health Sci. (2020) 14(1):20–3. PMID: 31983917 PMC6968880

[6] DeFrancesco CJ Blumberg TJ Chauvin NA Sankar WN. An improved method for measuring hip abduction in spica after surgical reduction for developmental dysplasia of the hip. J Child’s Orthop. (2017) 11(4):277–83. 10.1302/1863-2548.11.170038 PMC5584496 28904633

[7] Roposch A Stöhr KK Dobson M. The effect of the femoral head ossific nucleus in the treatment of developmental dysplasia of the hip. A meta-analysis. J Bone Joint Surg Am. (2009) 91(4):911–8. 10.2106/JBJS.H.00096 19339576

[8] Novais EN Hill MK Carry PM Heyn PC. Is age or surgical approach associated with osteonecrosis in patients with developmental dysplasia of the hip? A meta-analysis. Clin Orthop Relat Res. (2016) 474(5):1166–77. 10.1007/s11999-015-4590-5 26472583 PMC4814411

[9] Sankar WN Gornitzky AL Clarke NMP Herrera-Soto JA Kelley SP Matheney T Closed reduction for developmental dysplasia of the hip: early-term results from a prospective, multicenter cohort. J Pediatr Orthop. (2019) 39(3):111–8. 10.1097/BPO.0000000000000895 30730414 PMC6416015

[10] Wang YJ Yang F Wu QJ Pan SN Li LY. Association between open or closed reduction and avascular necrosis in developmental dysplasia of the hip A PRISMA-compliant meta-analysis of observational studies. Medicine. (2016) 95(29):e4276. 10.1097/MD.0000000000004276 27442664 PMC5265781

[11] Arneill M Cosgrove A Robinson E. Should closed reduction of the dislocated hip be attempted after failed Pavlik harness treatment in developmental dysplasia of the hip? Bone Jt Open. (2021) 2(8):594–8. 10.1302/2633-1462.28.BJO-2021-0088.R1 PMC8384449 34351213

[12] Liberati A Altman DG Tetzlaff J Mulrow C Gøtzsche PC Ioannidis JP The PRISMA statement for reporting systematic reviews and meta-analyses of studies that evaluate healthcare interventions: explanation and elaboration. Br Med J. (2009) 339(jul21 1):b2700. 10.1136/bmj.b2700 19622552 PMC2714672

[13] Kalamchi A MacEwen GD. Avascular necrosis following treatment of congenital dislocation of the hip. J Bone Joint Surg Am vol. (1980) 62(6):876–88. PMID: 7430175

[14] Bucholz RW Ogden JA. Patterns of Ischemic Necrosis of the Proximal Femur in Nonoperatively Treated Congenital Hip Disease. St Louis, MO: CV Mosby Company (1978). p. 43–63.

[15] Salter RB Kostuik J Dallas S. Avascular necrosis of the femoral head as a complication of treatment for congenital dislocation of the hip in young children: a clinical and experimental investigation. Can J Surg. (1969) 12(1):44–61. PMID: 5762671 5762671

[16] Stang A. Critical evaluation of the Newcastle-Ottawa scale for the assessment of the quality of nonrandomized studies in meta-analyses. Eur J Epidemiol. (2010) 25(9):603–5. 10.1007/s10654-010-9491-z 20652370

[17] Roposch A Odeh O Doria AS Wedge JH. The presence of an ossific nucleus does not protect against osteonecrosis after treatment of developmental dysplasia of the hip. Clin Orthop Relat Res. (2011) 469(10):2838–45. 10.1007/s11999-011-1801-6 21312075 PMC3171532

[18] Zhang G Li M Qu XY Cao YJ Liu X Luo C Efficacy of closed reduction for developmental dysplasia of the hip: midterm outcomes and risk factors associated with treatment failure and avascular necrosis. J Orthop Surg Res. (2020) 15(1):579. 10.1186/s13018-020-02098-3 33267908 PMC7709328

[19] Cheon JE Kim JY Choi YH Kim WS Cho TJ Yoo WJ. MRI Risk factors for development of avascular necrosis after closed reduction of developmental dysplasia of the hip: predictive value of contrast-enhanced MRI. PLoS One. (2021) 16(3 March):e0248701. 10.1371/journal.pone.0248701 33735261 PMC7971487

[20] Wu J Yuan Z Li J Zhu M Canavese F Fuxing X Does the vascular development of the femoral head correlate with the incidence of avascular necrosis of the proximal femoral epiphysis in children with developmental dysplasia of the hip treated by closed reduction? J Child’s Orthop. (2021) 15(4):395–401. 10.1302/1863-2548.15.210059 PMC8381394 34476030

[21] Kheiri S Tahririan MA Shahnaser S Ardakani MP. Avascular necrosis predictive factors after closed reduction in patients with developmental dysplasia of the hip. J Res Med Sci. (2023) 28(1):81. 10.4103/jrms.jrms_288_23 38292338 PMC10826850

[22] Tang Z Li R Lu C Ma N Xie R Kang X Risk factors for avascular necrosis of the femoral head after developmental hip dislocation reduction surgery and construction of nomogram prediction model. BMC Musculoskelet Disord. (2024) 25(1):464. 10.1186/s12891-024-07575-y 38877449 PMC11179329

[23] Zamzam M Bakarman KA Alaujan AA Houdane A AlKhayyal YA Al Zaid H Assessing avascular necrosis risk and outcomes after open reduction for developmental dysplasia of the hip in children. Cureus. (2024) 16(12):e75808. 10.7759/cureus.75808 39816296 PMC11734834

[24] Aǧuş H Ömeroǧlu H Uçar H Biçimoǧlu A Tümer Y. Evaluation of the risk factors of avascular necrosis of the femoral head in developmental dysplasia of the hip in infants younger than 18 months of Age1. J Pediatr Orthop B. (2002) 11(1):41–6. 10.1097/01202412-200201000-00007 11866080

[25] Pospischill R Weninger J Ganger R Altenhuber J Grill F. Does open reduction of the developmental dislocated hip increase the risk of osteonecrosis? Clin Orthop Relat Res. (2012) 470(1):250–60. 10.1007/s11999-011-1929-4 21643924 PMC3237975

[26] Schur MD Lee C Arkader A Catalano A Choi PD. Risk factors for avascular necrosis after closed reduction for developmental dysplasia of the hip. J Child’s Orthop. (2016) 10(3):185–92. 10.1007/s11832-016-0743-7 PMC4909658 27177477

[27] Liu YH Xu HW Li YQ Hong K Li JC Pereira B Effect of abduction on avascular necrosis of the femoral epiphysis in patients with late-detected developmental dysplasia of the hip treated by closed reduction: a MRI study of 59 hips. J Child’s Orthop. (2019) 13(5):438–44. 10.1302/1863-2548.13.190045 PMC6808074 31695810

[28] Apostolides M Gowda SR Roslee C Beamish B Burby N Richards RH. The presence of the ossific nucleus and its relation to avascular necrosis rates and the number of secondary procedures in late-presenting developmental dysplasia of the hip. J Pediatr Orthop B. (2021) 30(2):139–45. 10.1097/BPB.0000000000000767 32694427

[29] Bian Z Guo Y Lyu X Zhu Z Yang Z Wang Y. Risk factors for avascular necrosis after closed reduction for developmental dysplasia of the hip. J Pediatr Orthop. (2022) 42(9):467–73. 10.1097/BPO.0000000000002228 35948526 PMC9470038

[30] Bozkurt C Sarikaya B Sipahioğlu S Çetin BV Bekin Sarikaya PZ Kaptan AY Evaluation of avascular necrosis risk factors after closed reduction for developmental dysplasia of the hip before walking age. J Pediatr Orthop B. (2022) 31(3):237–41. 10.1097/BPB.0000000000000846 34116555

[31] Pang H Xu L Su F Li M Dang Y Wang B Association between ossific nucleus volume changes and postoperative avascular necrosis risk in children with developmental dysplasia of the hip. Sci Rep. (2024) 14(1):21363. 10.1038/s41598-024-72445-5 39266644 PMC11392927

[32] Wu QJ Li YY Lin YD Sun XW Ma HL Sun J Risk factor analysis of femoral avascular necrosis after operation for tonnis grade IV developmental dysplasia of the hip. Int Orthop. (2024) 48(4):1011–6. 10.1007/s00264-023-05996-3 37819386

[33] Qiu M Chen M Sun H Li D Cai Z Zhang W Avascular necrosis under different treatment in children with developmental dysplasia of the hip: a network meta-analysis. J Pediatr Orthop B. (2022) 31(4):319–26. 10.1097/BPB.0000000000000932 34751178

[34] Domos G Váncsa S Szeverényi C Agócs G Hegyi P Perge A Rates and risk factors for failure of reduction in closed reduction in developmental dysplasia of the hip: a systematic review and meta-analysis. Efort Open Rev. (2024) 9(9):908–22. 10.1530/EOR-24-0007 39222331 PMC11457818

[35] Walter SG Bornemann R Koob S Ossendorff R Placzek R. Closed reduction as therapeutic gold standard for treatment of congenital hip dislocation. Z Orthop Unfall. (2020) 158(5):475–80. 10.1055/a-0979-2346 31533169

[36] Yuan Z Li Y Hong K Wu J Canavese F Xu H. Poor delineation of labrum and acetabular surface on arthrogram is a predictor of early failure of closed reduction in children aged six to 24 months with developmental dysplasia of the hip. J Child’s Orthop. (2020) 14(5):372–8. 10.1302/1863-2548.14.200132 PMC7666794 33204344

[37] Castañeda P Masrouha KZ Ruiz CV Moscona-Mishy L. Outcomes following open reduction for late-presenting developmental dysplasia of the hip. J Child’s Orthop. (2018) 12(4):323–30. 10.1302/1863-2548.12.180078 PMC6090193 30154922

[38] Li Y Lin X Liu Y Li J Liu Y Pereira B Effect of age on radiographic outcomes of patients aged 6-24 months with developmental dysplasia of the hip treated by closed reduction. J Pediatr Orthopa B. (2020) 29(5):431–7. 10.1097/BPB.0000000000000672 31464797

[39] Wirth T Stratmann L Hinrichs F. Evolution of late presenting developmental dysplasia of the hip and associated surgical procedures after 14 years of neonatal ultrasound screening. J Bone Joint Surg Br Vol. (2004) 86(4):585–9. PMID: 15174558

[40] Yilar S Köse M Tuncer K Karsan O Topal M Ezirmik N. Impact of presence of ossific nucleus on results of closed reduction in treatment of developmental dysplasia of the hip (302 hips). J Pediatr Orthop B. (2021) 30(2):126–31. 10.1097/BPB.0000000000000752 32453121

[41] Segal LS Schneider DJ Berlin JM Bruno A Davis BR Jacobs CR. The contribution of the ossific nucleus to the structural stiffness of the capital femoral epiphysis: a porcine model for DDH. J Pediatr Orthop. (1999) 19(4):433–7. 10.1097/00004694-199907000-00003 10412989

[42] Sibiński M Synder M Domzalski M Grzegorzewski A. Risk factors for avascular necrosis after closed hip reduction in developmental dysplasia of the hip. Ortop Traumatol Rehabil. (2004) 6(1):60–6. PMID: 17676009

[43] Ramo BA De La Rocha A Sucato DJ Jo CH. A new radiographic classification system for developmental hip dysplasia is reliable and predictive of successful closed reduction and late pelvic osteotomy. J Pediatr Orthop. (2018) 38(1):16–21. 10.1097/BPO.0000000000000733 26866641

[44] Liu L Xiong QG Dai ZZ Ding J Wu ZK Dai Y. Closed reduction and Spica cast immobilization in patients aged 18 months and older with developmental dysplasia of the hip. Orthop Surg. (2025) 17(6):1702–9. 10.1111/os.70043 40260515 PMC12146125

[45] Sand A Tiderius CJ Düppe H Wenger D. The international hip dysplasia institute (IHDI) classification is more informative than the tönnis classification. Acta Radiol. (2023) 64(3):1103–8. 10.1177/02841851221110447 35758228

[46] Segal LS Boal DK Borthwick L Clark MW Localio AR Schwentker EP. Avascular necrosis after treatment of DDH: the protective influence of the ossific nucleus. J Pediatr Orthop. (1999) 19(2):177–84. 10.1097/00004694-199903000-00008 10088684

[47] Luhmann SJ Schoenecker PL Anderson AM Bassett GS. The prognostic importance of the ossific nucleus in the treatment of congenital dysplasia of the hip. J Bone Joint Surg Am Vol. (1998) 80(12):1719–27. 10.2106/00004623-199812000-00001 9875929

[48] Ogden JA. Changing patterns of proximal femoral vascularity. J Bone Joint Surg. (1974) 56(5):941–50. 10.2106/00004623-197456050-00007 4847241

[49] Niziol R Elvey M Protopapa E Roposch A. Association between the ossific nucleus and osteonecrosis in treating developmental dysplasia of the hip: updated meta-analysis. BMC Musculoskelet Disord. (2017) 18(1):165. 10.1186/s12891-017-1468-6 28427427 PMC5397826

[50] Carney BT Clark D Minter CL. Is the absence of the ossific nucleus prognostic for avascular necrosis after closed reduction of developmental dysplasia of the hip? J Surg Orthop Adv. (2004) 13(1):24–9. PMID: 15055492

