# Supplementary material for: Risk factors for avascular necrosis of the femoral head after surgical treatment of developmental dysplasia of the hip in children: a systematic review and meta-analysis
Source: Front Pediatr. 2026 Apr 13;14:1811138. doi: 10.3389/fped.2026.1811138 (PMC13111955; doi:10.3389/fped.2026.1811138)
Supplement: Supplementary file 1 [file Supplementaryfile1.docx]

Pubmed

| Search number | Query | Sort By | Filters | Search Details | Results |
| --- | --- | --- | --- | --- | --- |
| 1 | Developmental Dysplasia of the Hip[MeSH Terms] | | | "developmental dysplasia of the hip"[MeSH Terms] | 8,913 |
| 2 | (((((((((Developmental Dysplasia of the Hip[Title/Abstract]) OR (Developmental Hip Dysplasia[Title/Abstract])) OR (Developmental Hip Dysplasias[Title/Abstract])) OR (Dysplasia, Developmental Hip[Title/Abstract])) OR (Hip Dysplasia, Developmental[Title/Abstract])) OR (Hip Dislocation, Developmental[Title/Abstract])) OR (Developmental Hip Dislocations[Title/Abstract])) OR (Dislocation, Developmental Hip[Title/Abstract])) OR (Developmental Hip Dislocation[Title/Abstract])) OR (DDH[Title/Abstract]) | | | "developmental dysplasia of the hip"[Title/Abstract] OR "developmental hip dysplasia"[Title/Abstract] OR "developmental hip dysplasias"[Title/Abstract] OR (("Dysplasia"[All Fields] OR "Dysplasias"[All Fields]) AND "developmental hip"[Title/Abstract]) OR "hip dysplasia developmental"[Title/Abstract] OR "hip dislocation developmental"[Title/Abstract] OR "developmental hip dislocations"[Title/Abstract] OR "dislocation developmental hip"[Title/Abstract] OR "developmental hip dislocation"[Title/Abstract] OR "DDH"[Title/Abstract] | 4,596 |
| 3 | (Developmental Dysplasia of the Hip[MeSH Terms]) OR ((((((((((Developmental Dysplasia of the Hip[Title/Abstract]) OR (Developmental Hip Dysplasia[Title/Abstract])) OR (Developmental Hip Dysplasias[Title/Abstract])) OR (Dysplasia, Developmental Hip[Title/Abstract])) OR (Hip Dysplasia, Developmental[Title/Abstract])) OR (Hip Dislocation, Developmental[Title/Abstract])) OR (Developmental Hip Dislocations[Title/Abstract])) OR (Dislocation, Developmental Hip[Title/Abstract])) OR (Developmental Hip Dislocation[Title/Abstract])) OR (DDH[Title/Abstract])) | | | "developmental dysplasia of the hip"[MeSH Terms] OR ("developmental dysplasia of the hip"[Title/Abstract] OR "developmental hip dysplasia"[Title/Abstract] OR "developmental hip dysplasias"[Title/Abstract] OR (("Dysplasia"[All Fields] OR "Dysplasias"[All Fields]) AND "developmental hip"[Title/Abstract]) OR "hip dysplasia developmental"[Title/Abstract] OR "hip dislocation developmental"[Title/Abstract] OR "developmental hip dislocations"[Title/Abstract] OR "dislocation developmental hip"[Title/Abstract] OR "developmental hip dislocation"[Title/Abstract] OR "DDH"[Title/Abstract]) | 11,177 |
| 4 | Femur Head Necroses[MeSH Terms] | | | "femur head necrosis"[MeSH Terms] | 9,118 |
| 5 | ((((((((((((Femur Head Necroses[Title/Abstract]) OR (Head Necrosis, Femur[Title/Abstract])) OR (Necrosis, Femur Head[Title/Abstract])) OR (Necrosis, Avascular, of Femur Head[Title/Abstract])) OR (Avascular Necrosis Of Femoral Head, Primary[Title/Abstract])) OR (Avascular Necrosis of Femur Head[Title/Abstract])) OR (Avascular Necrosis of the Femoral Head[Title/Abstract])) OR (Femoral Head, Avascular Necrosis Of[Title/Abstract])) OR (Femoral avascular necrosis[Title/Abstract])) OR (Ischemic Necrosis Of Femoral Head[Title/Abstract])) OR (Aseptic Necrosis of Femur Head[Title/Abstract])) OR (Necrosis, Aseptic, of Femur Head[Title/Abstract])) OR (Avascular Necrosis[Title/Abstract]) | | | "femur head necroses"[Title/Abstract] OR (("Head"[MeSH Terms] OR "Head"[All Fields]) AND "necrosis femur"[Title/Abstract]) OR (("necrose"[All Fields] OR "necrosed"[All Fields] OR "necrosi"[All Fields] OR "necrosing"[All Fields] OR "Necrosis"[MeSH Terms] OR "Necrosis"[All Fields] OR "Necroses"[All Fields]) AND "femur head"[Title/Abstract]) OR ((("necrose"[All Fields] OR "necrosed"[All Fields] OR "necrosi"[All Fields] OR "necrosing"[All Fields] OR "Necrosis"[MeSH Terms] OR "Necrosis"[All Fields] OR "Necroses"[All Fields]) AND ("Avascular"[All Fields] OR "avascularity"[All Fields] OR "avascularized"[All Fields])) AND "of femur head"[Title/Abstract]) OR (("femur head necrosis"[MeSH Terms] OR ("Femur"[All Fields] AND "Head"[All Fields] AND "Necrosis"[All Fields]) OR "femur head necrosis"[All Fields] OR ("Avascular"[All Fields] AND "Necrosis"[All Fields] AND "Femoral"[All Fields] AND "Head"[All Fields]) OR "avascular necrosis of femoral head"[All Fields]) AND "Primary"[Title/Abstract]) OR "avascular necrosis of femur head"[Title/Abstract] OR "avascular necrosis of the femoral head"[Title/Abstract] OR "femoral head avascular necrosis of"[Title/Abstract] OR "femoral avascular necrosis"[Title/Abstract] OR "ischemic necrosis of femoral head"[Title/Abstract] OR "aseptic necrosis of femur head"[Title/Abstract] OR ((("necrose"[All Fields] OR "necrosed"[All Fields] OR "necrosi"[All Fields] OR "necrosing"[All Fields] OR "Necrosis"[MeSH Terms] OR "Necrosis"[All Fields] OR "Necroses"[All Fields]) AND ("Aseptic"[All Fields] OR "aseptical"[All Fields] OR "aseptics"[All Fields])) AND "of femur head"[Title/Abstract]) OR "avascular necrosis"[Title/Abstract] | 9,749 |
| 6 | (Femur Head Necroses[MeSH Terms]) OR (((((((((((((Femur Head Necroses[Title/Abstract]) OR (Head Necrosis, Femur[Title/Abstract])) OR (Necrosis, Femur Head[Title/Abstract])) OR (Necrosis, Avascular, of Femur Head[Title/Abstract])) OR (Avascular Necrosis Of Femoral Head, Primary[Title/Abstract])) OR (Avascular Necrosis of Femur Head[Title/Abstract])) OR (Avascular Necrosis of the Femoral Head[Title/Abstract])) OR (Femoral Head, Avascular Necrosis Of[Title/Abstract])) OR (Femoral avascular necrosis[Title/Abstract])) OR (Ischemic Necrosis Of Femoral Head[Title/Abstract])) OR (Aseptic Necrosis of Femur Head[Title/Abstract])) OR (Necrosis, Aseptic, of Femur Head[Title/Abstract])) OR (Avascular Necrosis[Title/Abstract])) | | | "femur head necrosis"[MeSH Terms] OR ("femur head necroses"[Title/Abstract] OR (("Head"[MeSH Terms] OR "Head"[All Fields]) AND "necrosis femur"[Title/Abstract]) OR (("necrose"[All Fields] OR "necrosed"[All Fields] OR "necrosi"[All Fields] OR "necrosing"[All Fields] OR "Necrosis"[MeSH Terms] OR "Necrosis"[All Fields] OR "Necroses"[All Fields]) AND "femur head"[Title/Abstract]) OR ((("necrose"[All Fields] OR "necrosed"[All Fields] OR "necrosi"[All Fields] OR "necrosing"[All Fields] OR "Necrosis"[MeSH Terms] OR "Necrosis"[All Fields] OR "Necroses"[All Fields]) AND ("Avascular"[All Fields] OR "avascularity"[All Fields] OR "avascularized"[All Fields])) AND "of femur head"[Title/Abstract]) OR (("femur head necrosis"[MeSH Terms] OR ("Femur"[All Fields] AND "Head"[All Fields] AND "Necrosis"[All Fields]) OR "femur head necrosis"[All Fields] OR ("Avascular"[All Fields] AND "Necrosis"[All Fields] AND "Femoral"[All Fields] AND "Head"[All Fields]) OR "avascular necrosis of femoral head"[All Fields]) AND "Primary"[Title/Abstract]) OR "avascular necrosis of femur head"[Title/Abstract] OR "avascular necrosis of the femoral head"[Title/Abstract] OR "femoral head avascular necrosis of"[Title/Abstract] OR "femoral avascular necrosis"[Title/Abstract] OR "ischemic necrosis of femoral head"[Title/Abstract] OR "aseptic necrosis of femur head"[Title/Abstract] OR ((("necrose"[All Fields] OR "necrosed"[All Fields] OR "necrosi"[All Fields] OR "necrosing"[All Fields] OR "Necrosis"[MeSH Terms] OR "Necrosis"[All Fields] OR "Necroses"[All Fields]) AND ("Aseptic"[All Fields] OR "aseptical"[All Fields] OR "aseptics"[All Fields])) AND "of femur head"[Title/Abstract]) OR "avascular necrosis"[Title/Abstract]) | 15,769 |
| 7 | Risk Factors[MeSH Terms] | | | "risk factors"[MeSH Terms] | 1,033,303 |
| 8 | ((((((((((((((((((((Risk Factors[Title/Abstract]) OR (Factor, Risk[Title/Abstract])) OR (Risk Factor[Title/Abstract])) OR (Population at Risk[Title/Abstract])) OR (Populations at Risk[Title/Abstract])) OR (Risk Scores[Title/Abstract])) OR (Risk Score[Title/Abstract])) OR (Score,Risk[Title/Abstract])) OR (Risk Factor Scores[Title/Abstract])) OR (Risk Factor Score[Title/Abstract])) OR (Score, Risk Factor[Title/Abstract])) OR (Health Correlates[Title/Abstract])) OR (Correlates, Health[Title/Abstract])) OR (Social Risk Factors[Title/Abstract])) OR (Factor, Social Risk[Title/Abstract])) OR (Factors, Social Risk[Title/Abstract])) OR (Risk Factor, Social[Title/Abstract])) OR (Risk Factors, Social[Title/Abstract])) OR (Social Risk Factor[Title/Abstract])) OR (Risk[Title/Abstract] AND Outcomes[Title/Abstract])) OR (Risk[Title/Abstract]) | | | "risk factors"[Title/Abstract] OR "factor risk"[Title/Abstract] OR "risk factor"[Title/Abstract] OR "population at risk"[Title/Abstract] OR "populations at risk"[Title/Abstract] OR "risk scores"[Title/Abstract] OR "risk score"[Title/Abstract] OR "score risk"[Title/Abstract] OR "risk factor scores"[Title/Abstract] OR "risk factor score"[Title/Abstract] OR "score risk factor"[Title/Abstract] OR "health correlates"[Title/Abstract] OR "correlates health"[Title/Abstract] OR "social risk factors"[Title/Abstract] OR (("Factor"[All Fields] OR "factor s"[All Fields] OR "Factors"[All Fields]) AND "social risk"[Title/Abstract]) OR "factors social risk"[Title/Abstract] OR "risk factor social"[Title/Abstract] OR "risk factors social"[Title/Abstract] OR "social risk factor"[Title/Abstract] OR ("Risk"[Title/Abstract] AND "Outcomes"[Title/Abstract]) OR "Risk"[Title/Abstract] | 3,138,354 |
| 9 | (Risk Factors[MeSH Terms]) OR (((((((((((((((((((((Risk Factors[Title/Abstract]) OR (Factor, Risk[Title/Abstract])) OR (Risk Factor[Title/Abstract])) OR (Population at Risk[Title/Abstract])) OR (Populations at Risk[Title/Abstract])) OR (Risk Scores[Title/Abstract])) OR (Risk Score[Title/Abstract])) OR (Score,Risk[Title/Abstract])) OR (Risk Factor Scores[Title/Abstract])) OR (Risk Factor Score[Title/Abstract])) OR (Score, Risk Factor[Title/Abstract])) OR (Health Correlates[Title/Abstract])) OR (Correlates, Health[Title/Abstract])) OR (Social Risk Factors[Title/Abstract])) OR (Factor, Social Risk[Title/Abstract])) OR (Factors, Social Risk[Title/Abstract])) OR (Risk Factor, Social[Title/Abstract])) OR (Risk Factors, Social[Title/Abstract])) OR (Social Risk Factor[Title/Abstract])) OR (Risk[Title/Abstract] AND Outcomes[Title/Abstract])) OR (Risk[Title/Abstract])) | | | "risk factors"[MeSH Terms] OR ("risk factors"[Title/Abstract] OR "factor risk"[Title/Abstract] OR "risk factor"[Title/Abstract] OR "population at risk"[Title/Abstract] OR "populations at risk"[Title/Abstract] OR "risk scores"[Title/Abstract] OR "risk score"[Title/Abstract] OR "score risk"[Title/Abstract] OR "risk factor scores"[Title/Abstract] OR "risk factor score"[Title/Abstract] OR "score risk factor"[Title/Abstract] OR "health correlates"[Title/Abstract] OR "correlates health"[Title/Abstract] OR "social risk factors"[Title/Abstract] OR (("Factor"[All Fields] OR "factor s"[All Fields] OR "Factors"[All Fields]) AND "social risk"[Title/Abstract]) OR "factors social risk"[Title/Abstract] OR "risk factor social"[Title/Abstract] OR "risk factors social"[Title/Abstract] OR "social risk factor"[Title/Abstract] OR ("Risk"[Title/Abstract] AND "Outcomes"[Title/Abstract]) OR "Risk"[Title/Abstract]) | 3,460,580 |
| 10 | (((Developmental Dysplasia of the Hip[MeSH Terms]) OR ((((((((((Developmental Dysplasia of the Hip[Title/Abstract]) OR (Developmental Hip Dysplasia[Title/Abstract])) OR (Developmental Hip Dysplasias[Title/Abstract])) OR (Dysplasia, Developmental Hip[Title/Abstract])) OR (Hip Dysplasia, Developmental[Title/Abstract])) OR (Hip Dislocation, Developmental[Title/Abstract])) OR (Developmental Hip Dislocations[Title/Abstract])) OR (Dislocation, Developmental Hip[Title/Abstract])) OR (Developmental Hip Dislocation[Title/Abstract])) OR (DDH[Title/Abstract]))) AND ((Femur Head Necroses[MeSH Terms]) OR (((((((((((((Femur Head Necroses[Title/Abstract]) OR (Head Necrosis, Femur[Title/Abstract])) OR (Necrosis, Femur Head[Title/Abstract])) OR (Necrosis, Avascular, of Femur Head[Title/Abstract])) OR (Avascular Necrosis Of Femoral Head, Primary[Title/Abstract])) OR (Avascular Necrosis of Femur Head[Title/Abstract])) OR (Avascular Necrosis of the Femoral Head[Title/Abstract])) OR (Femoral Head, Avascular Necrosis Of[Title/Abstract])) OR (Femoral avascular necrosis[Title/Abstract])) OR (Ischemic Necrosis Of Femoral Head[Title/Abstract])) OR (Aseptic Necrosis of Femur Head[Title/Abstract])) OR (Necrosis, Aseptic, of Femur Head[Title/Abstract])) OR (Avascular Necrosis[Title/Abstract])))) AND ((Risk Factors[MeSH Terms]) OR (((((((((((((((((((((Risk Factors[Title/Abstract]) OR (Factor, Risk[Title/Abstract])) OR (Risk Factor[Title/Abstract])) OR (Population at Risk[Title/Abstract])) OR (Populations at Risk[Title/Abstract])) OR (Risk Scores[Title/Abstract])) OR (Risk Score[Title/Abstract])) OR (Score,Risk[Title/Abstract])) OR (Risk Factor Scores[Title/Abstract])) OR (Risk Factor Score[Title/Abstract])) OR (Score, Risk Factor[Title/Abstract])) OR (Health Correlates[Title/Abstract])) OR (Correlates, Health[Title/Abstract])) OR (Social Risk Factors[Title/Abstract])) OR (Factor, Social Risk[Title/Abstract])) OR (Factors, Social Risk[Title/Abstract])) OR (Risk Factor, Social[Title/Abstract])) OR (Risk Factors, Social[Title/Abstract])) OR (Social Risk Factor[Title/Abstract])) OR (Risk[Title/Abstract] AND Outcomes[Title/Abstract])) OR (Risk[Title/Abstract]))) | | | ("developmental dysplasia of the hip"[MeSH Terms] OR ("developmental dysplasia of the hip"[Title/Abstract] OR "developmental hip dysplasia"[Title/Abstract] OR "developmental hip dysplasias"[Title/Abstract] OR (("Dysplasia"[All Fields] OR "Dysplasias"[All Fields]) AND "developmental hip"[Title/Abstract]) OR "hip dysplasia developmental"[Title/Abstract] OR "hip dislocation developmental"[Title/Abstract] OR "developmental hip dislocations"[Title/Abstract] OR "dislocation developmental hip"[Title/Abstract] OR "developmental hip dislocation"[Title/Abstract] OR "DDH"[Title/Abstract])) AND ("femur head necrosis"[MeSH Terms] OR ("femur head necroses"[Title/Abstract] OR (("Head"[MeSH Terms] OR "Head"[All Fields]) AND "necrosis femur"[Title/Abstract]) OR (("necrose"[All Fields] OR "necrosed"[All Fields] OR "necrosi"[All Fields] OR "necrosing"[All Fields] OR "Necrosis"[MeSH Terms] OR "Necrosis"[All Fields] OR "Necroses"[All Fields]) AND "femur head"[Title/Abstract]) OR ((("necrose"[All Fields] OR "necrosed"[All Fields] OR "necrosi"[All Fields] OR "necrosing"[All Fields] OR "Necrosis"[MeSH Terms] OR "Necrosis"[All Fields] OR "Necroses"[All Fields]) AND ("Avascular"[All Fields] OR "avascularity"[All Fields] OR "avascularized"[All Fields])) AND "of femur head"[Title/Abstract]) OR (("femur head necrosis"[MeSH Terms] OR ("Femur"[All Fields] AND "Head"[All Fields] AND "Necrosis"[All Fields]) OR "femur head necrosis"[All Fields] OR ("Avascular"[All Fields] AND "Necrosis"[All Fields] AND "Femoral"[All Fields] AND "Head"[All Fields]) OR "avascular necrosis of femoral head"[All Fields]) AND "Primary"[Title/Abstract]) OR "avascular necrosis of femur head"[Title/Abstract] OR "avascular necrosis of the femoral head"[Title/Abstract] OR "femoral head avascular necrosis of"[Title/Abstract] OR "femoral avascular necrosis"[Title/Abstract] OR "ischemic necrosis of femoral head"[Title/Abstract] OR "aseptic necrosis of femur head"[Title/Abstract] OR ((("necrose"[All Fields] OR "necrosed"[All Fields] OR "necrosi"[All Fields] OR "necrosing"[All Fields] OR "Necrosis"[MeSH Terms] OR "Necrosis"[All Fields] OR "Necroses"[All Fields]) AND ("Aseptic"[All Fields] OR "aseptical"[All Fields] OR "aseptics"[All Fields])) AND "of femur head"[Title/Abstract]) OR "avascular necrosis"[Title/Abstract])) AND ("risk factors"[MeSH Terms] OR ("risk factors"[Title/Abstract] OR "factor risk"[Title/Abstract] OR "risk factor"[Title/Abstract] OR "population at risk"[Title/Abstract] OR "populations at risk"[Title/Abstract] OR "risk scores"[Title/Abstract] OR "risk score"[Title/Abstract] OR "score risk"[Title/Abstract] OR "risk factor scores"[Title/Abstract] OR "risk factor score"[Title/Abstract] OR "score risk factor"[Title/Abstract] OR "health correlates"[Title/Abstract] OR "correlates health"[Title/Abstract] OR "social risk factors"[Title/Abstract] OR (("Factor"[All Fields] OR "factor s"[All Fields] OR "Factors"[All Fields]) AND "social risk"[Title/Abstract]) OR "factors social risk"[Title/Abstract] OR "risk factor social"[Title/Abstract] OR "risk factors social"[Title/Abstract] OR "social risk factor"[Title/Abstract] OR ("Risk"[Title/Abstract] AND "Outcomes"[Title/Abstract]) OR "Risk"[Title/Abstract])) | 193 |

WOS

| Entitlements | # | Search Query | Database | Results |
| --- | --- | --- | --- | --- |
| - WOS.IC: 1993 to 2025 - WOS.CCR: 1985 to 2025 - WOS.SCI: 1996 to 2025 - WOS.AHCI: 1996 to 2025 - WOS.ESCI: 2020 to 2025 - WOS.ISTP: 2002 to 2025 - WOS.SSCI: 1996 to 2025 - WOS.ISSHP: 2002 to 2025 | 1 | TS=(Developmental Dysplasia of the Hip) OR TS=(Developmental Hip Dysplasia) OR TS=(Developmental Hip Dysplasias) OR TS=(Dysplasia, Developmental Hip) OR TS=(Hip Dysplasia, Developmental) OR TS=(Hip Dislocation, Developmental) OR TS=(Developmental Hip Dislocations) OR TS=(Dislocation, Developmental Hip) OR TS=(Developmental Hip Dislocation) OR TS=(DDH) | Web of Science Core Collection | 5957 |
| - WOS.IC: 1993 to 2025 - WOS.CCR: 1985 to 2025 - WOS.SCI: 1996 to 2025 - WOS.AHCI: 1996 to 2025 - WOS.ESCI: 2020 to 2025 - WOS.ISTP: 2002 to 2025 - WOS.SSCI: 1996 to 2025 - WOS.ISSHP: 2002 to 2025 | 2 | TS=(Femur Head Necroses) OR TS=(Head Necrosis, Femur) OR TS=(Necrosis, Femur Head) OR TS=(Necrosis, Avascular, of Femur Head) OR TS=(Avascular Necrosis Of Femoral Head, Primary) OR TS=(Avascular Necrosis of Femur Head ) OR TS=(Avascular Necrosis of the Femoral Head) OR TS=(Femoral Head, Avascular Necrosis Of) OR TS=(Femoral avascular necrosis) OR TS=(Ischemic Necrosis Of Femoral Head) OR TS=(Aseptic Necrosis of Femur Head) OR TS=(Necrosis, Aseptic, of Femur Head) OR TS=(Avascular Necrosis) | Web of Science Core Collection | 9432 |
| - WOS.IC: 1993 to 2025 - WOS.CCR: 1985 to 2025 - WOS.SCI: 1996 to 2025 - WOS.AHCI: 1996 to 2025 - WOS.ESCI: 2020 to 2025 - WOS.ISTP: 2002 to 2025 - WOS.SSCI: 1996 to 2025 - WOS.ISSHP: 2002 to 2025 | 3 | TS=(Risk Factors) OR TS=(Factor, Risk) OR TS=(Risk Factor) OR TS=(Population at Risk) OR TS=(Populations at Risk) OR TS=(Risk Scores) OR TS=(Risk Score) OR TS=(Score,Risk) OR TS=(Risk Factor Scores) OR TS=(Risk Factor Score) OR TS=(Score, Risk Factor) OR TS=(Health Correlates) OR TS=(Correlates, Health) OR TS=(Social Risk Factors) OR TS=(Factor, Social Risk) OR TS=(Factors, Social Risk) OR TS=(Risk Factor, Social) OR TS=(Risk Factors, Social) OR TS=(Social Risk Factor) OR TS=(Risk and Outcomes) OR TS=(Risk) | Web of Science Core Collection | 4674938 |
| - WOS.IC: 1993 to 2025 - WOS.CCR: 1985 to 2025 - WOS.SCI: 1996 to 2025 - WOS.AHCI: 1996 to 2025 - WOS.ESCI: 2020 to 2025 - WOS.ISTP: 2002 to 2025 - WOS.SSCI: 1996 to 2025 - WOS.ISSHP: 2002 to 2025 | 4 | #1 and #2 and #3 | Web of Science Core Collection | 172 |

Embase

| No. | Query | Results |
| --- | --- | --- |
| #49 | #12 AND #26 AND #48 | 207 |
| #48 | #27 OR #28 OR #29 OR #30 OR #31 OR #32 OR #33 OR #34 OR #35 OR #36 OR #37 OR #38 OR #39 OR #40 OR #41 OR #42 OR #43 OR #44 OR #45 OR #46 OR #47 | 4904639 |
| #47 | 'risk':ab,ti | 4492607 |
| #46 | 'risk and outcomes':ab,ti | 759 |
| #45 | 'social risk factor':ab,ti | 142 |
| #44 | 'risk factors, social':ab,ti | 161 |
| #43 | 'frisk factor, social':ab,ti | 0 |
| #42 | 'factors, social risk':ab,ti | 10 |
| #41 | 'factor, social risk':ab,ti | 0 |
| #40 | 'social risk factors':ab,ti | 1580 |
| #39 | 'correlates, health':ab,ti | 13 |
| #38 | 'health correlates':ab,ti | 686 |
| #37 | 'score, risk factor':ab,ti | 17 |
| #36 | 'risk factor score':ab,ti | 308 |
| #35 | 'risk factor scores':ab,ti | 155 |
| #34 | 'score,risk':ab,ti | 1436 |
| #33 | 'risk score':ab,ti | 55645 |
| #32 | 'risk scores':ab,ti | 26729 |
| #31 | 'populations at risk':ab,ti | 3960 |
| #30 | 'population at risk':ab,ti | 6302 |
| #29 | 'risk factor':ab,ti | 439383 |
| #28 | 'factor, risk':ab,ti | 367 |
| #27 | 'risk factor'/exp | 1535921 |
| #26 | #13 OR #14 OR #15 OR #16 OR #17 OR #18 OR #19 OR #20 OR #21 OR #22 OR #23 OR #24 OR #25 | 13111 |
| #25 | 'necrosis, aseptic, of femur head':ab,ti | 0 |
| #24 | 'aseptic necrosis of femur head':ab,ti | 6 |
| #23 | 'ischemic necrosis of femoral head':ab,ti | 44 |
| #22 | 'femoral avascular necrosis':ab,ti | 24 |
| #21 | 'femoral head, avascular necrosis of':ab,ti | 5 |
| #20 | 'avascular necrosis of the femoral head':ab,ti | 1978 |
| #19 | 'avascular necrosis of femur head':ab,ti | 12 |
| #18 | 'avascular necrosis of femoral head, primary':ab,ti | 0 |
| #17 | 'necrosis, avascular, of femur head':ab,ti | 0 |
| #16 | 'necrosis, femur head':ab,ti | 1 |
| #15 | 'head necrosis, femur':ab,ti | 0 |
| #14 | 'femur head necroses':ab,ti | 11 |
| #13 | 'avascular necrosis'/exp | 12131 |
| #12 | #1 OR #2 OR #3 OR #4 OR #5 OR #6 OR #7 OR #8 OR #9 OR #10 OR #11 | 11232 |
| #11 | 'ddh':ab,ti | 3364 |
| #10 | 'developmental hip dislocation':ab,ti | 62 |
| #9 | 'dislocation, developmental hip':ab,ti | 1 |
| #8 | 'developmental hip dislocations':ab,ti | 8 |
| #7 | 'hip dislocation, developmental':ab,ti | 6 |
| #6 | 'hip dysplasia, developmental':ab,ti | 4 |
| #5 | 'dysplasia, developmental hip':ab,ti | 1 |
| #4 | 'developmental hip dysplasias':ab,ti | 1 |
| #3 | 'developmental hip dysplasia':ab,ti | 469 |
| #2 | 'developmental dysplasia of the hip':ab,ti | 3410 |
| #1 | 'hip dysplasia'/exp | 9247 |

Cochrane

ID Search Hits

#1 MeSH descriptor: [Developmental Dysplasia of the Hip] explode all trees 163

#2 (Developmental Dysplasia of the Hip):ti,ab,kw OR (Developmental Hip Dysplasia):ti,ab,kw OR (Developmental Hip Dysplasias):ti,ab,kw OR (Dysplasia, Developmental Hip):ti,ab,kw OR (Hip Dysplasia, Developmental):ti,ab,kw 222

#3 (Hip Dislocation, Developmental):ti,ab,kw OR (Developmental Hip Dislocations):ti,ab,kw OR (Dislocation, Developmental Hip):ti,ab,kw OR (Developmental Hip Dislocation):ti,ab,kw OR (DDH):ti,ab,kw 197

#4 #1 or #2 or #3 323

#5 MeSH descriptor: [Femur Head Necrosis] explode all trees 197

#6 (Femur Head Necroses):ti,ab,kw OR (Head Necrosis, Femur):ti,ab,kw OR (Necrosis, Femur Head):ti,ab,kw OR (Necrosis, Avascular, of Femur Head):ti,ab,kw OR (Avascular Necrosis Of Femoral Head, Primary):ti,ab,kw 379

#7 (Avascular Necrosis of Femur Head):ti,ab,kw OR (Avascular Necrosis of the Femoral Head):ti,ab,kw OR (Femoral Head, Avascular Necrosis Of):ti,ab,kw OR (Femoral avascular necrosis):ti,ab,kw OR (Ischemic Necrosis Of Femoral Head):ti,ab,kw 300

#8 (Aseptic Necrosis of Femur Head):ti,ab,kw OR (Necrosis, Aseptic, of Femur Head):ti,ab,kw OR (Avascular Necrosis):ti,ab,kw 526

#9 #5 or #6 pr #7 or #8 671

#10 MeSH descriptor: [Risk Factors] explode all trees 37696

#11 (Risk Factors):ti,ab,kw OR (Factor, Risk):ti,ab,kw OR (Risk Factor):ti,ab,kw OR (Population at Risk):ti,ab,kw OR (Populations at Risk):ti,ab,kw 159882

#12 (Risk Scores):ti,ab,kw OR (Risk Score):ti,ab,kw OR (Score,Risk):ti,ab,kw OR (Risk Factor Scores):ti,ab,kw OR (Risk Factor Score):ti,ab,kw 56469

#13 (Score, Risk Factor):ti,ab,kw OR (Health Correlates):ti,ab,kw OR (Correlates, Health):ti,ab,kw OR (Social Risk Factors):ti,ab,kw OR (Factor, Social Risk):ti,ab,kw 16574

#14 (Factors, Social Risk):ti,ab,kw OR (Risk Factor, Social):ti,ab,kw OR (Risk Factors, Social):ti,ab,kw OR (Social Risk Factor):ti,ab,kw OR (Risk and Outcomes):ti,ab,kw 87813

#15 (Risk):ti,ab,kw 314196

#16 #10 or #11 or #12 or #13 or #14 or #15 315318

#17 #4 and #9 and #16 9
