# Supplementary material for: Risk factors for avascular necrosis of the femoral head after surgical treatment of developmental dysplasia of the hip in children: a systematic review and meta-analysis
Source: Front Pediatr. 2026 Apr 13;14:1811138. doi: 10.3389/fped.2026.1811138 (PMC13111955; doi:10.3389/fped.2026.1811138)
Supplement: Supplementary file 2 [file Datasheet1.docx]

Records identified from: Databases (n = 579)

PubMed(n=193)

Embase(n=207)

Cochrane library(n=7)

Web of science(n=172)

Records removed *before screening*:

Duplicate records removed (n = 187)

Records marked as ineligible by automation tools (n =21)

Records removed for other reasons (n =9)

**Identification**

Records after removal of duplicates(n = 362)

Records excluded by Reading the title and abstract (n =321)

Reports excluded(n=23):

There was no relevant outcome (n =17)

Combined with other interventions (n =4)

Data unavailable (n =2)

Full-text articles evaluated for eligibility(n = 41)

**Screening**

Exclude cases that have not undergone AVN classification (n =2)

Reports assessed for eligibility

(n = 18)

Studies included in quantitative synithesis (Meta-analysis)

(n = 16)

**Included**
